# Three-dimensional structure of printer toner visualized using cryogenic X-ray diffraction imaging tomography

**DOI:** 10.1107/S1600577525008008

**Published:** 2025-10-16

**Authors:** Kosei Harada, Yuki Takayama, Masayoshi Nakasako

**Affiliations:** a Department of Physics, Faculty of Science and Technology Keio University 3-14-1 Hiyoshi, Kohoku-ku Yokohama Kanagawa223-8522 Japan; b RIKEN SPring-8 Center, 1-1-1 Kohto, Sayo, Sayo-gun, Hyogo679-5148, Japan; c International Center for Synchrotron Radiation Innovation Smart Tohoku University 468-1 Aramaki Aza-Aoba Aoba-ku Sendai980-8572 Japan; RIKEN SPring-8 Center, Japan

**Keywords:** cryogenic X-ray diffraction imaging tomography, non-crystalline particle, three-dimensional structure, printer toner, triboelectrification

## Abstract

Printer toner is a fine powder material essential for fixing digital information on paper during the electrostatic digital printing process. To achieve fine printing, toner particles are composed of colored pigments, dyes, polymers, charge control agents and other surfactants. Toner particles have approximate dimensions of 5–10 µm. Therefore, the distribution of the constituents cannot be observed by transmission electron microscopy. In this study, we visualized the three-dimensional structure of a 5 µm-sized printer toner particle using cryogenic X-ray diffraction imaging tomography. A particle adsorbed on a silicon nitride membrane was rotated in the angular range from −78° to +78° against the direction of the incident X-rays at an angular step of 0.5°. From the 313 diffraction patterns collected, we reconstructed the three-dimensional electron density distribution of the toner particle at a resolution of 141 nm. The particle was wedge-shaped. The electron density distribution inside the particle was non-uniform. The high-electron-density regions were distributed near the surface. These formed a V-shaped structure at the tip of the wedge. With the help of powder diffraction, the high-electron-density regions were interpreted as microcrystals of silicon dioxide. Silicon dioxide crystals function as cleavage sites for toner particles prepared by the milling method. Based on the present results, we discuss the implications of the structure and methods to visualize it at a higher resolution.

## Abstract 2

The nonuniform internal structure of a printer-toner particle with an approximate size of 5 µm was visualized at a resolution of 141 nm using cryogenic X-ray diffraction imaging tomography.

## 1. Introduction

Printer toner is a fine powder material essential for forming digital information such as texts and images on paper in electrostatic digital printing processes that use photocopiers, scanners and printer devices (Verma *et al.*, 2018 ▸). In electrostatic digital printing using a light-emitting diode (LED) (Seol *et al.*, 2018 ▸), a device called a drum (or roller) is charged according to a digital image projected by the LED light. The charged drum collects toner particles and transfers them to paper through triboelectrification, that is the process by which materials become electrically charged after contact with different materials (Ko *et al.*, 2021 ▸). The toner particles are then melted by heat, and the colored pigments/dyes in the particles are bonded permanently to the paper.

For achieving the printing process, toner particles with approximate dimensions of 5–10 µm are made of several constituents such as colored pigments/dyes, polymers, charge control agents, paraffin wax, and other surfactants (Verma *et al.*, 2018 ▸). A set of cyan, magenta, yellow and black toners enable us to obtain a significantly large color variation in the printing. Polymers function as media for other constituents. These have melting temperatures of approximately 340 K for thermally transferring colored pigments/dyes to paper (Ishihara *et al.*, 1998 ▸). Charge control agents provide the electrostatic characteristics of toner particles necessary for triboelectrification (Verma *et al.*, 2018 ▸). Surfactants increase the adherence of toner particles to paper surfaces. Paraffin wax prevents it from sticking to the heated drum (Verma *et al.*, 2018 ▸). The parameters affecting the efficiencies of printing processes are the type of constituents, their distribution inside the toner particles, the size and surface shape of the toner particles, *etc*. For example, finer-quality printed media require smaller toner particles; for example, to achieve a resolution of 1200 dots per inch, toner particles with sizes of approximately 3 µm may be necessary (Ataeefard, 2014 ▸). To improve the resolution of printing and understand the triboelectrification mechanism in printing, it is necessary to determine the distribution of the constituents in the toner particles.

Spectroscopic methods and mass spectrometry have been applied to study the different features of powder toners necessary for their identification in the manufacture of toners in forensic sciences (Pirela *et al.*, 2015 ▸). Transmission electron microscopy (TEM) (Radermacher, 1988 ▸) is used to determine the various constituents of the powder toners. However, owing to the strong interactions between free electrons and the electrostatic potential produced by constituent atoms, whole toner particles with thicknesses of 5–10 µm are almost electron-opaque. Although TEM requires sectioning of particles with a thickness of less than 100 nm, the degradation of particles by physicochemical treatment may be infeasible to identify. Therefore, sectioning and chemical treatments should be prevented to visualize the intrinsic internal structures of all toner particles.

X-ray diffraction imaging tomography at cryogenic temperature (cXDI-TM) is used to study the three-dimensional (3D) structures of non-crystalline particles under reduced radiation damage conditions (Kobayashi *et al.*, 2018 ▸; Nakasako, 2018 ▸). X-rays with short wavelengths have high penetration power for thick objects. Therefore, cXDI-TM is suitable for investigating the internal three-dimensional (3D) structures of noncrystalline particles with a size of several micrometres, without requiring sectioning. In a cXDI-TM experiment, a single non-crystalline particle whose temperature is maintained near 77 K is rotated against the direction of the incident X-rays with a high transverse coherence (Kobayashi *et al.*, 2018 ▸).

A 3D electron density map of the specimen particles is reconstructed from a set of projection maps retrieved from the diffraction patterns collected in the tomography experiment. cXDI-TM experiments have successfully demonstrated the potential for the study of the internal structure of whole non-crystalline particles such as cells with dimensions in the micrometre range (Rodriguez *et al.*, 2015 ▸; Kobayashi *et al.*, 2018 ▸; Nakasako, 2018 ▸; Nakasako *et al.*, 2020 ▸; Sung *et al.*, 2021 ▸).

In this study, we applied cXDI-TM to visualize the internal structure of a toner particle. As the toner particle was composed of polymers, cryogenic experiments were essential to reduce the radiation damage of polymer constituents (Storp, 1985 ▸; Miura, 2000 ▸). The reconstructed 3D map revealed the nonuniform distribution of the constituents and the characteristic distribution pattern of the high-density regions. These results may contribute to the development of finer toner particles for higher resolution printing and the examination of toxicological effects (Getzlaff *et al.*, 2019 ▸).

## 2. Experimental procedures and structural analyses

### 2.1. Scanning electron microscopy, X-ray powder diffraction and trace element analysis

Prior to the cXDI-TM experiment, magenta toner particles (TNR-C4FM2, OKI, Japan) were characterized using scanning electron microscopy (SEM), X-ray powder diffraction and energy-dispersive X-ray fluorescence spectroscopy (EDXRF).

The SEM images were obtained using a TM3000 scanning electron microscope (Hitachi High-Tech, Tokyo, Japan). A trace element analysis based on EDXRF was performed using an M4 TORNADO (Bruker, Billerica, USA). An Rh source was operated at 50 kV and 60 µA.

The powder diffraction profiles were collected using a D8 ADVANCE diffractometer (Bruker, Billerica, MA, USA) operated at 40 kV and 40 mA. Cu *K*α radiation (1.5418 Å; 10 Å = 1 nm) was selected. The scan step and scan speed were 0.01° and 0.25° min^−1^, respectively. From the full width at half-maximum (FWHM, *w*_B_) of the diffraction peak at a Bragg angle (θ_B_), the average size (*H*) of the microcrystals contained in the toner particles was estimated using the following equation (Leoni, 2019 ▸),
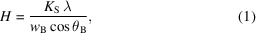
where *K*_S_ is the Sherrer constant and λ is the X-ray wavelength. Assuming that the microcrystals are spherical with cubic symmetry, *K*_S_ is 0.94.

### 2.2. Specimen preparation for cryogenic tomography experiment

We used a specimen disk (SiRN-7.5-200-3.0-100, Silson, Warwickshire, UK). Therein, a 100 nm-thick Si_3_N_4_ membrane (SiN membrane) was spanned on a silicon frame of 7.5 mm × 7.5 mm with a 3 mm × 3 mm window at the center. The SiN membrane was coated with a carbon layer of approximate thickness 10 nm using a CADE-E vacuum evaporator (MEIWAFOSIS Co. Ltd, Tokyo, Japan) to conduct the electrons produced by the X-ray irradiation.

The specimens for the cXDI-TM experiments were prepared using a custom-built specimen preparation chamber (Takayama & Nakasako, 2012 ▸; Takayama & Yonekura, 2016 ▸; Kobayashi *et al.*, 2016 ▸). The chamber was fixed on the stage of an inverted optical microscope (Nikon, Tokyo, Japan) and filled with high-humidity air supplied by a moist air generator HUM-1 (Rigaku, Tokyo, Japan) (Takayama & Nakasako, 2012 ▸). Purchased magenta toner was suspended in 70%(*v*/*v*) ethanol (Wako, Japan) solution and dispersed on a cover glass (Matsunami, Osaka, Japan) placed in the specimen-preparation chamber. We observed that the surfaces of the toner particles on the cover glass became sticky when the humidity in the specimen preparation chamber was set to approximately 50% relative humidity (RH). Then, a single particle on the cover glass was attached to a tip of a TransferTip glass capillary with an inner diameter of 15 µm (ES, Eppendorf, Leipzig Germany) by manipulating it using a motor-controlled micro-manipulator (SURUGA SEIKI, Tokyo, Japan), and transferred near the center of the SiN membrane of the specimen disk using the TransferTip. The specimens were prepared as rapidly as feasible. We did not observe any variations in the shape or size of the toner particles under an optical microscope. After the evaporation of the remaining ethanol solution, the position of the particle from the edges of the Si frame of the specimen disk was recorded using the microscope.

### 2.3. cXDI-TM experiment

We performed a cXDI-TM experiment using our custom-built diffraction apparatus KOTOBUKI-1 (Nakasako *et al.*, 2013 ▸; Kobayashi *et al.*, 2018 ▸; Nakasako, 2018 ▸; Nakasako *et al.*, 2020 ▸) at the second experimental hutch of BL29XUL of SPring-8. The X-ray wavelength was tuned to 0.2254 nm (corresponding to an X-ray photon energy of 5.500 keV) using a fixed-exit double-crystal monochromator. The higher-order harmonics of the selected X-rays were reduced significantly using a Pt-coated flat double-mirror system placed downstream of the monochromator.

A pinhole with a diameter of 38 µm was placed at 1950 mm upstream of the specimen position. It produced an X-ray beam with full spatial coherence within a radius of 7 µm at the specimen position (Kobayashi *et al.*, 2018 ▸; Nakasako, 2018 ▸). To reduce the parasitic scattering from beamline optics, we set two silicon frames with beveled 100 µm × 100 µm and 120 µm × 120 µm windows at 420 mm and 15 mm upstream of the specimen position, respectively. A PIN photodiode (S3590-09, Hamamatsu Photonics KK, Hamamatsu, Japan) was used to measure the intensity of the incident X-ray beam. The diffraction patterns were recorded using an EIGER detector (DECTRIS, Baden-Daettwil, Switzerland) placed at 5.95 m downstream of the specimen position. A beamstop of 1 mm × 1 mm was placed 0.30 m upstream of the detector.

The specimen disk carrying the toner particle was transported to the specimen stage inside the vacuum chamber of the diffraction apparatus using a specially designed carrier and the load-lock chamber of the apparatus. The specimen disk was initially set such that the SiN membrane was normal to the direction of the incident X-rays. Then, the nominal rotation angle was defined to be 0°. The toner particle was set into the irradiation area of the incident X-ray beam, and one diffraction pattern was collected at ambient temperature under an irradiation dose of 4.3 × 10^5^ Gy.

After the exposure, liquid nitro­gen was supplied to a cryogenic pot fixed at the top of a four-axis goniometer using an automatic liquid nitro­gen supplier system controlled by a MODEL LM-510 Liquid Cryogen Level Monitor (Cryomagnetics Inc., Tennesse, USA). The toner particle was cooled through thermal conduction to the cooled pot. As the polymer rods connecting the goniometer and the pot gradually shrinks by approximately 254 µm for 10 h, the data collection was started 11 h after the supply of liquid nitro­gen. The temperature of the stage was maintained at 77 ± 1 K by evaporation-cooling effect during the experiment.

A series of diffraction patterns for structural analysis were collected automatically in the angular range from −78° to +78° in a step of 0.5°. The diffraction patterns could not be collected in the angular ranges of −90° to −78° and +78° to +90°; this was because the silicon frame of the specimen disk hindered both incident and diffracted X-rays. At each angle, a single diffraction pattern of the toner particle on the SiN membrane was recorded by a 180 s exposure, after optimally adjusting the particle position against the center of the incident X-ray beam. In addition, a diffraction pattern of the SiN membrane was recorded by a 180 s exposure for an area at a distance of over 100 µm from the toner particle along the rotation axis.

The total irradiation dose for the toner particle was estimated using the following equation proposed in a previous study (Jiang *et al.*, 2010 ▸),
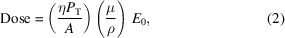
where *E*_0_ is the photon energy of the incident X-rays. The total number of X-ray photons *P*_T_ is calculated using the photon flux (3.7 × 10^10^ photons s^−1^) and the total exposure time including positional adjustment at each angle. η is the fraction of the incident X-rays (20.5%) for the toner particle with an approximate cross section of 5 µm × 5 µm. μ/ρ is the mass absorption coefficient of the toner particle, assumed to be 1.8 m^2^ kg^−1^.

### 2.4. Data processing

Prior to data processing, we masked several hot spots displaying 3.0 × 10^5^ counts s^−1^ pixel^−1^ and the gap regions between the detector panels. The net diffraction pattern at each angle was obtained by subtracting the diffraction pattern of the SiN membrane area from that of the toner particle. The gap regions of the detector panels were filled with patterns in the symmetry mate regions.

The beam center position in each diffraction pattern was searched at a 0.1 pixel resolution for efficient convergence in the subsequent phase-retrieval (PR) calculations. Then, for a resolution shell from 8.56 to 11.07 µm^−1^, we minimized *R*_merge_, defined as
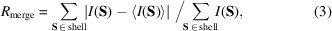
where *I*(**S**) is the intensity at the scattering vector **S**. The length of **S** is defined using the scattering/diffraction angle, 2θ, and the wavelength of the X-rays, λ, as *S* = 2sinθ/λ. *I*(−**S**) is the intensity of the symmetry mate with respect to the beam center position in the search calculation; 〈*I*(*S*)〉 is their average. For each beam center position assumed in the search, the diffraction intensity of each pixel was calculated using the bicubic interpolation method. From our experience, an *R*_merge_ value of less than 0.3 is favorable for the highest-resolution shell. Then, the degree of centrosymmetry of each diffraction pattern in a region of interest (ROI) was also evaluated using a metric defined as (Fujioka *et al.*, 2008 ▸; Sekiguchi *et al.*, 2014 ▸)
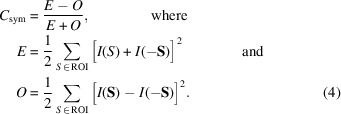
*C*_sym_ values higher than 0.8 are favorable for phase retrieval calculation.

### 2.5. Phase retrieval calculation

In this study, we trimmed diffraction patterns at a resolution of 7.10 µm^−1^ (corresponding to 141 nm in real space). For the diffraction from a particle with an approximate size of 5 µm, a Ewald sphere of 0.2254 nm X-rays can be approximated as a plane up to a resolution of 29.9 µm^−1^ (Oroguchi & Nakasako, 2013 ▸). The diffraction amplitudes can then be assumed to be the modulus of the structure factor of the electron-density map projected along the direction of the incident X-ray beam.

For each diffraction pattern, we performed 300 PR calculations using the hybrid-input–output (Fienup, 1982 ▸) and shrink-wrap (Marchesini *et al.*, 2003 ▸) algorithms (Kodama & Nakasako, 2011 ▸; Oroguchi & Nakasako, 2013 ▸; Yoshida *et al.*, 2024 ▸). Each calculation started from an initial map with random electron densities different from those in the other PR calculations. The threshold used in the shrink-wrap algorithm was determined prior to starting the 300 PR calculations. The obtained 300 PR maps were superimposed to a selected reference map using a pattern-matching algorithm.

To select the most probable pair from the 300 PR maps, we used a similarity score (Sekiguchi *et al.*, 2017 ▸), defined as
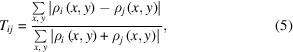
where ρ_*i*_(*x*, *y*) is the electron density at a pixel position (*x*, *y*) of the *i*th map. In our experience, the most probable maps display the lowest similarity scores (Sekiguchi *et al.*, 2017 ▸; Takayama & Nakasako, 2024 ▸; Yoshida *et al.*, 2024 ▸). In addition, for one of the pairs, we calculated the crystallographic *R*-factor, *R*_F_, defined as
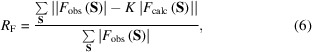
where |*F*_obs_(**S**)| and |*F*_calc_(**S**)| denote the experimentally observed structure amplitudes and those calculated from the most probable PR map, respectively. *K* is a scale factor determined to equalize the sum of |*F*_calc_(**S**)| with that of |*F*_obs_(**S**)|. For each diffraction pattern, as the pair of maps displaying the smallest similarity score (0.006–0.025) were almost the same, one of the pair was selected for the subsequent structure analysis.

To automatically select the most probable map pair throughout the 313 PR maps, we first determined a map pair at 0.0° for use as a reference in selecting map pairs at the adjacent angles, *i.e.* −0.5° and +0.5°. The selected PR map pair at −0.5° (+0.5°) was then used as a reference for the PR calculations at −1.0° (+1.0°). This procedure was conducted automatically and sequentially until the diffraction patterns at both the end angles.

### 2.6. Reconstruction of three-dimensional electron density map

The selected PR maps were normalized and scaled with respect to the sum of the electron densities. The maps at ±0.5° were superimposed to minimize the similarity score against the map at 0.0°. Then, the maps at ±1.0° were superimposed on the maps at ±0.5°. This procedure was applied successively to the maps at adjacent rotation angles.

A 3D map of the toner particle was reconstructed from the selected and superimposed 313 PR maps using a simultaneous iterative reconstruction technique (SIRT) (Gilbert, 1972 ▸). In the SIRT calculation, we minimized the following residual as
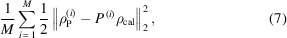
where ρ_cal_ is the electron density to be reconstructed, and 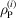 and *P*^(*i*)^ are the electron density and the projection-matrix of the *i*th projection, respectively. In the steepest descent method used in the minimization, the update equation is written as
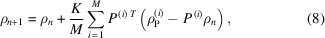
where *K* is a scale factor and *T* indicates the transpose operation. The calculation was carried out until the residual converged. In this study, a SIRT iteration of 93 times was sufficient for the convergence.

After the convergence of the SIRT calculation, we evaluated the similarity score between the map reprojected from the 3D map and the PR map at each rotation angle. Additionally, we calculated the crystallographic *R*-factor between the diffraction amplitudes of the reprojection map and the observed diffraction amplitudes.

## 3. Results

### 3.1. Preliminary characterization of toner particles

**Figure 1:**
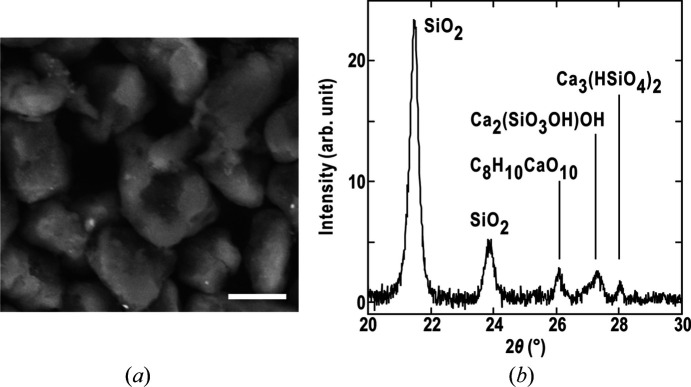
(*a*) SEM image of magenta toner particles. The scale bar is 2 µm. (*b*) X-ray powder diffraction profile and feasible assignment of the diffraction peaks.

Fig. 1 ▸(*a*) shows an SEM image of the magenta toner particles. The size of the toner particles ranged from 5 to 10 µm. The particles exhibited both planar and curved surfaces. Images of the particles helped in examining whether the 3D structure analysis was successful. A trace element analysis indicated that the magenta toner particles contained silicon (composition ratio of 62%) and calcium (26%) as the major components.

In the X-ray powder diffraction profiles [Fig. 1 ▸(*b*)], we observed two Bragg peaks at diffraction angles of 21.5° and 23.9° for the silicon dioxide crystal. The other three compounds likely originated from calcium hydrogen malate hydrate and calcium silicate hydroxide crystals. The size of the silicon dioxide microcrystals was estimated to be 28 nm from the FWHM values using equation (1). The size of the silicon dioxide crystal was three times that in a Lyreco toner (10.1 nm) and two times that in a Hewlett-Packard toner (16.5 nm) (Getzlaff *et al.*, 2019 ▸).

### 3.2. Diffraction patterns

**Figure 2:**
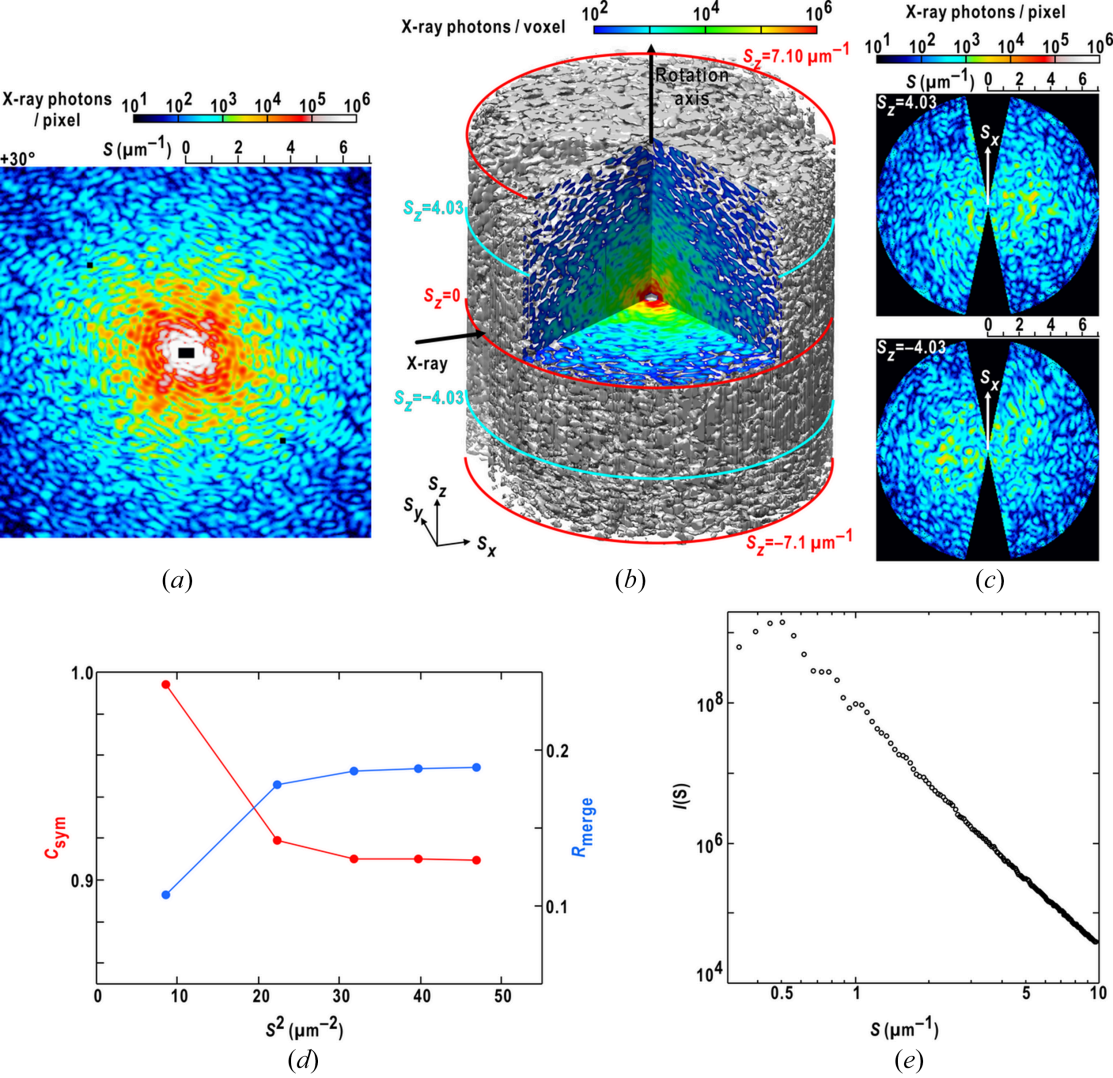
Diffraction data collected in the cXDI-TM experiment. (*a*) A representative diffraction pattern of the toner particle at +30°. (*b*) Distribution of diffraction intensity in reciprocal space up to a resolution of 7.1 µm^−1^. (*c*) Comparison of diffraction patterns in a pair of symmetry-related sections at ±4.03 µm^−1^. (*d*) Resolution-dependent variations in *C*_sym_ and *R*_merge_ plotted against the square of the scattering vector length to equalize the volumes of the resolution shells. (*e*) X-ray diffraction profile up to a resolution of 10 µm^−1^ calculated by summing the radial average of each diffraction pattern. Panel (*b*) was prepared using *ChimeraX* (Pettersen *et al.*, 2021 ▸).

We collected 313 diffraction patterns in the angular range from −78° to +78° at an angular step of 0.5° (Fig. 2 ▸, and Movie S1 of the supporting information). We observed speckle peaks with a signal-to-noise ratio higher than four up to a resolution of 15 µm^−1^ (corresponding to 65 nm in real space).

Each diffraction pattern is composed of well separated speckle peaks. This reflects the full spatial coherence of the incident X-ray beam [Fig. 2 ▸(*a*)]. The reciprocal of the smallest speckle peak size (approximately 0.25 µm^−1^) corresponds to an approximate size of the particle (4 µm). In the 3D distribution of the diffraction intensity from the 313 diffraction patterns [Fig. 2 ▸(*b*)], centrosymmetry was likely satisfied. This was demonstrated by the selected *S*_*z*_-sections along the rotation axis [Fig. 2 ▸(*c*)] and was evaluated quantitatively by *C*_sym_ and *R*_merge_ [Fig. 2 ▸(*d*)]. The variation ranges of the two metrics were acceptable for the use in the structure analysis.

A radially averaged diffraction intensity profile summed over the 313 diffraction patterns displayed no diffraction maxima in the resolution from 0.4 to 10 µm^−1^. This observation implies the absence of any regularly arranged electron densities when viewed at a resolution of 100 nm.

### 3.3. Three-dimensional map

**Figure 3:**
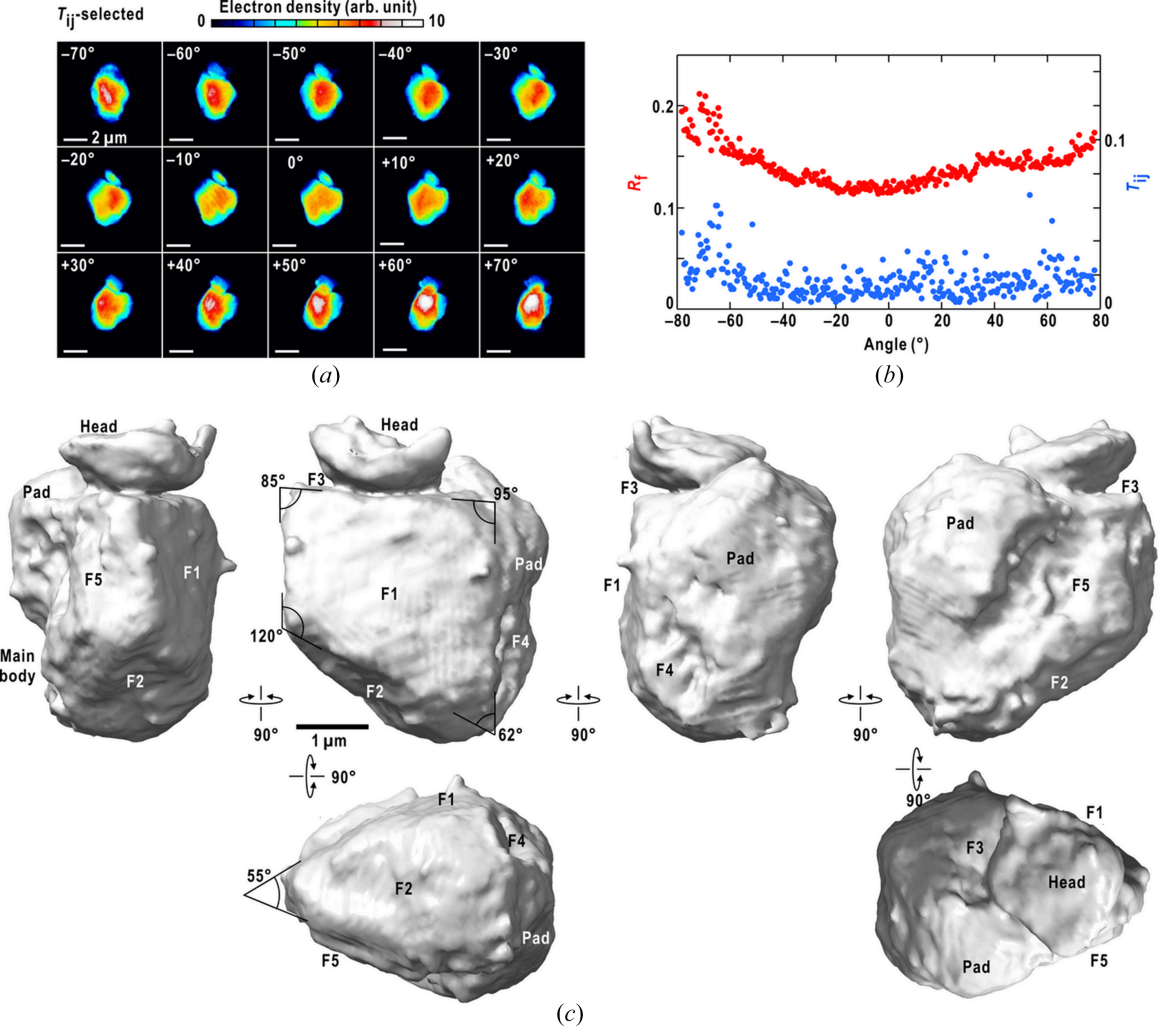
Three-dimensional reconstruction of the electron density distribution in the toner particle. (*a*) A set of projection electron density maps retrieved from the diffraction patterns at 10° intervals of the rotation. The maximum resolution used in the phase-retrieval calculation was 141 nm. The scale bar is 2 µm. (*b*) Crystallographic *R*-factor and similarity score of the selected map at each rotation angle. (*c*) Six views of the surface structures of the toner particle contoured at the 0.18 level of the relative electron density. The names of the parts and surfaces of the main body and certain edge-angle values are labeled (see the main text). This panel was prepared using *ChimeraX* (Pettersen *et al.*, 2021 ▸).

A set of PR maps was retrieved from the 313 diffraction patterns trimmed at a resolution of 7.1 µm^−1^ [Fig. 3 ▸(*a*)]. The similarity scores were smaller than 0.05. The crystallographic *R*-factors were in the range 0.11–0.21 [Fig. 3 ▸(*b*)]. At rotation angles with larger apparent SiN membrane thicknesses against the direction of the incident X-ray, the PR maps yielded higher *R*-factor values. This was likely because of the incomplete incorporation of background patterns in the present data processing procedure.

A 3D map of the toner particle was reconstructed from the 313 retrieved maps at a resolution of 141 nm [Fig. 3 ▸(*c*)]. The particle had approximate dimensions of 4.2 µm × 4.9 µm × 3.2 µm and roughly comprised three parts: ‘main body’, ‘pad’ and ‘head’. The main body had an approximate size of 4.2 µm × 3.9 µm × 2.5 µm and a wedge-shape, which was formed by five semi-planar surfaces, F1–F5. The trapeze-shaped surface F1 was the largest and smoothest among the five surfaces. The edge angles were approximately 60°, 90° and 120°. The angle between the surfaces F1 and F5 was 55°. Unlike F1–F4, F5 had a rugged surface composed of shallow crevasses. The presence of edges and semi-planar surfaces of the main body was consistent with the shapes of the toner particles observed in the SEM image [Fig. 1 ▸(*a*)]. The pad had dimensions of 2.3 µm × 4.1 µm × 0.9 µm. It was composed of a semi-spherical part attached to surfaces F3 and F5, and likely combined with F4. The head, with a disk-shape of dimensions 1.0 µm × 1.9 µm × 2.2 µm, was likely formed independently from the main body and adsorbed on surface F3

**Figure 4:**
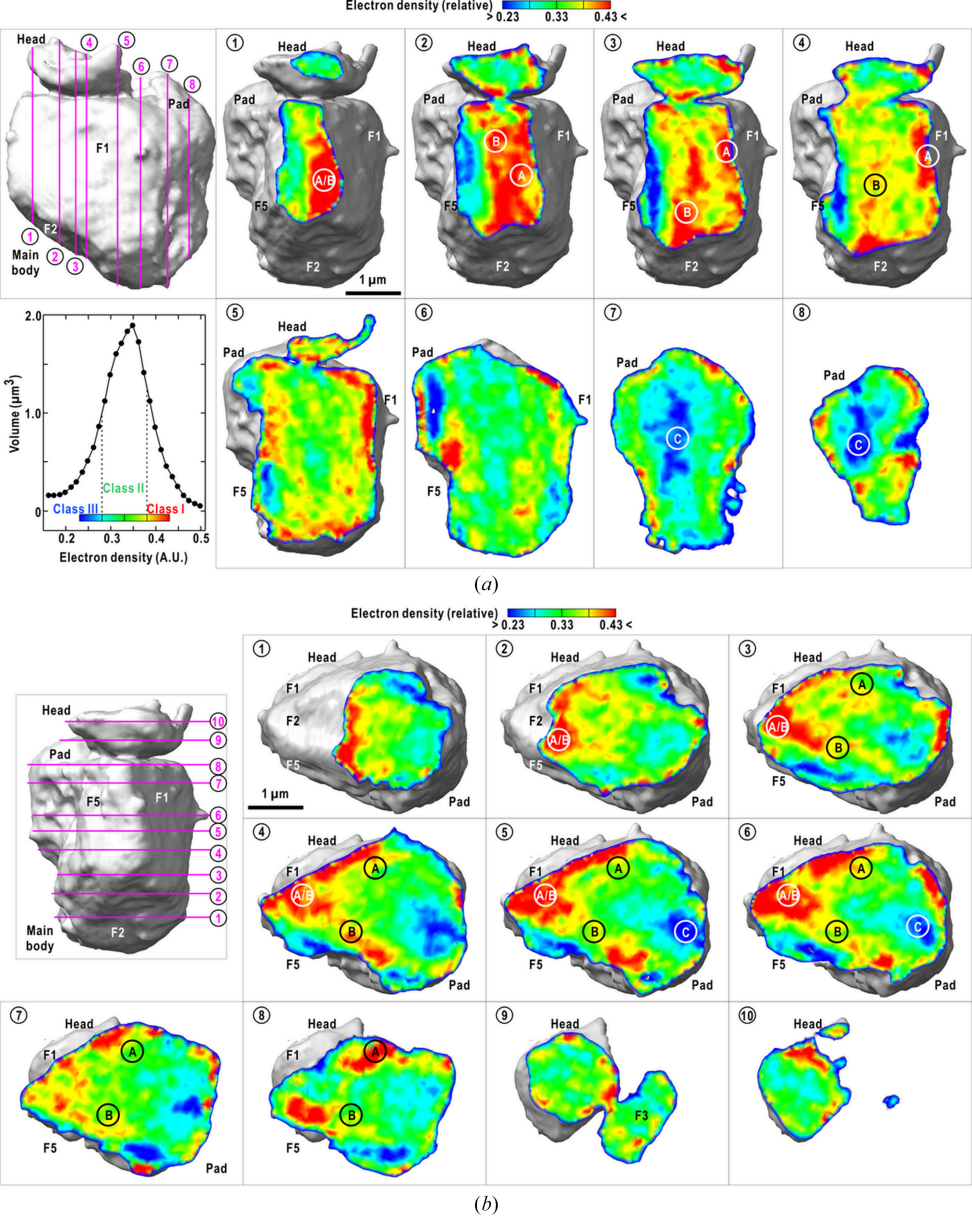
Cross-sectional views of electron density distribution inside the particle along the directions normal to surface F1 (*a*) and the wedge (*b*). The plot at the lower left in panel (*a*) shows the volume fraction at each electron density level. The names of the surfaces and characteristic electron density distributions (‘A’, ‘B’, ‘A/B’ and ‘C’) are labeled. The panels were prepared using *ChimeraX* (Pettersen *et al.*, 2021 ▸).

The electron density distribution inside the toner particle was non-uniform (Fig. 4 ▸). With respect to the electron density level, we classified the internal structures into three classes, I–III [see the inset plot in Fig. 4 ▸(*a*)]. This indicates that the constituents of the toner particles were combined non-uniformly and formed sub-micrometre-sized aggregates.

Class I with density higher than 0.38 occupied approximately 21% (4.13 µm^3^) of the entire volume (20.1 µm^3^). It was predominantly distributed in the vicinity of the surfaces rather than the central region [cross-sectional views 1–3 of Fig. 4 ▸(*a*) and 3–6 of Fig. 4 ▸(*b*)]. The largest regions of class I at the tip of the wedge are designated ‘A’, ‘B’ and ‘A/B’ in cross-sectional views 1–4 of Fig. 4 ▸(*a*). These formed a V-shaped structure under surfaces F1, F5 and F2 [cross-sectional views 3–6 in Fig. 4 ▸(*b*)]. Although region A was extended as a thin layer with a thickness of 0.2–0.3 µm, region B comprised several high-density peaks with approximate sizes of 0.2–0.3 µm [cross-sectional view 3 of Fig. 4 ▸(*a*) and 3–7 of Fig. 4 ▸(*b*)]. When viewed at a resolution of 100 nm, these high-density peaks are distributed randomly, as anticipated from the diffraction profile without peaks or enhancements [Fig. 2 ▸(*e*)]. In addition to the wedge, class I was distributed at the interface between the head and surface F3.

Class II with a density between 0.28 and 0.38 occupied 63% (12.69 µm^3^) of the total volume. It was distributed over the main body and head [cross-sectional views 5–6 of Figs. 4 ▸(*a*) and 4 ▸(*b*)]. The difference in the electron density levels implies that the constituents of class II were likely different from those of class I. The surfaces formed by class II had fewer edges than those of class I.

Class III had a density lower than 0.28 and occupied 16% (3.32 µm^3^) of the total volume. It predominantly occupied the central part of the pad and the pad-main body interface [region ‘C’ in cross-sectional views 7–8 of Fig. 4 ▸(*a*) and 4–7 of Fig. 4 ▸(*b*)]. Few class III densities were observed in the vicinity of the class I regions. This implies that the constituents of classes I and III were difficult to combine.

## 4. Discussion

In this study, we visualized the structure of a toner particle at a resolution of 141 nm using cXDI-TM. The reconstructed electron density revealed a nonuniform electron density distribution inside the particle. Here, we describe the implications of the structure and the means to extend its resolution.

### 4.1. Structure of toner particle

Polymerization and milling methods are used to prepare toner powders (Ishihara *et al.*, 1998 ▸). In the polymerization method, toner particles are produced in a dispersion media. In the milling method, a piece of mixed toner constituents is mechanically broken into powder. As the toner particle had edge surfaces F1, F2 and F5, these were likely manufactured using the milling method.

The constituents used in the toner are concealed from users. Therefore, we speculate on the relationship between the three electron density classes and constituents. The toner particle contained silicon dioxide microcrystals with an approximate size of 28 nm [Fig. 1 ▸(*b*)]. As the silicon dioxide microcrystals and their aggregates had the highest electron density among the constituents, silicon dioxide crystals likely were the major component of the class I density (Fig. 4 ▸). In addition, the cleavage planes of the silicon dioxide crystal in the hexagonal lattice system can explain the angle values of the four edges and the wedge [Fig. 3 ▸(*c*)]. Therefore, silicon dioxide crystals may function as cleavage sites to produce toner particles by the milling method. The exposure of silicon dioxide crystals may be advantageous for efficient charge transfer during printing and the association of toner particles, as observed between the head and surface F3 [sections 2–5 of Fig. 4 ▸(*a*)]. Therefore, a 3D map of the toner particle may provide a structural basis for understanding the triboelectrification mechanism between toner and paper.

As the composition of the three electron density classes in the head is similar to that in the main body, further milling of the main body may provide finer particles similar to the head. In the milling method, finer particles may be produced by increasing the concentration of silicon dioxide crystals in a mixture of constituents.

### 4.2. Necessity of cryogenic experiment

**Figure 5:**
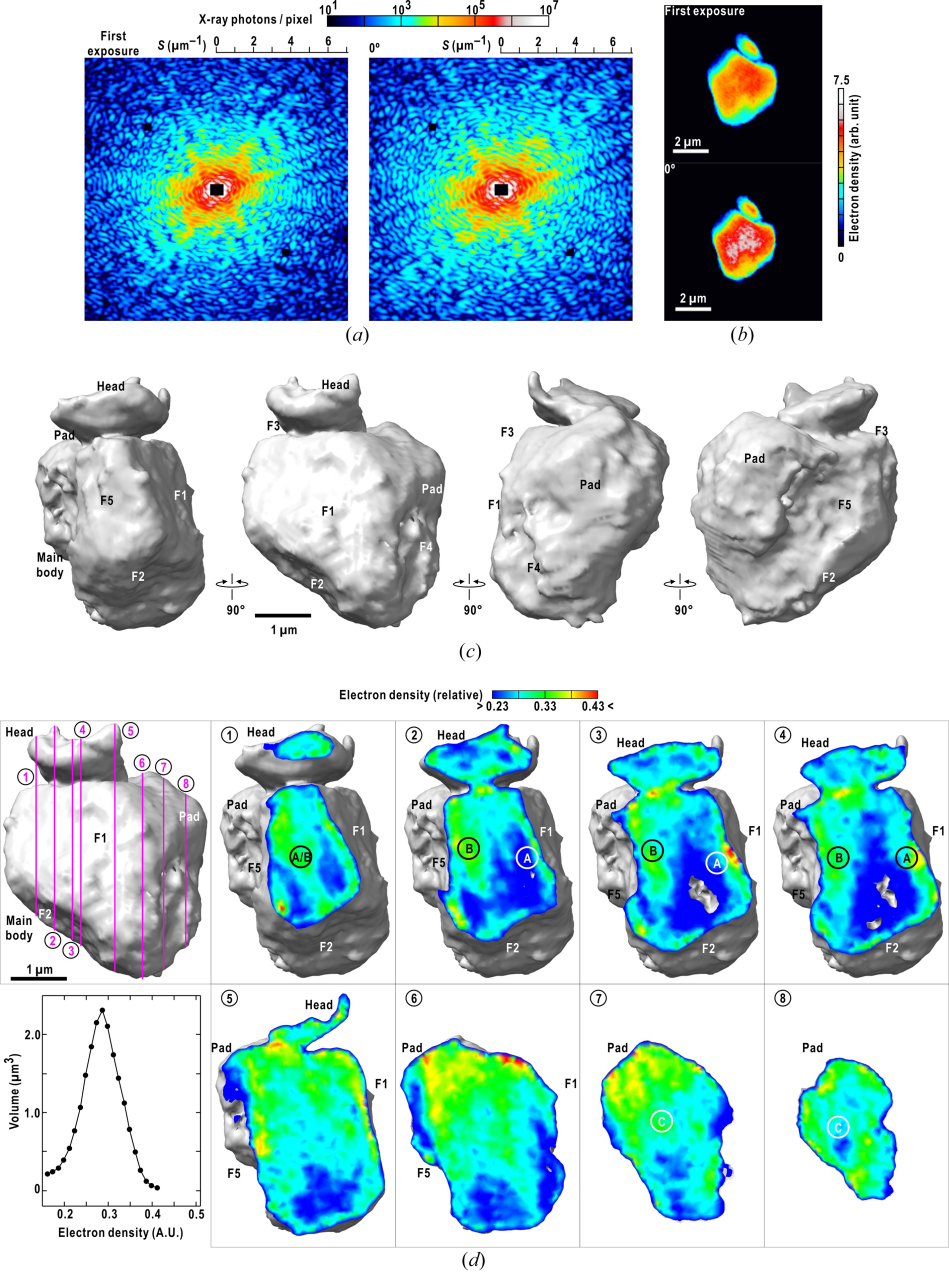
(*a*) Diffraction patterns collected at 298 K (left) and at 77 K (right) after the irradiation of 5.5 × 10^7^ Gy. (*b*) Projection electron density maps retrieved from the diffraction patterns in panel (*a*). (*c*) Surface structure of a 3D map reconstructed from a set of diffraction patterns of the warmed-and-cooled toner particle. The diffraction patterns were collected from −80° to +80° at an angular step of 0.5°. The X-ray flux was 3.1 × 10^10^ photons s^−1^ and the exposure time was 100 s. Therefore, the diffraction intensities were scaled to those of the first experiment. (*d*) Sectional views of the 3D map. Panels (*c*) and (*d*) were prepared using *ChimeraX* (Pettersen *et al.*, 2021 ▸).

In this study, we kept the temperature of the toner particle to suppress the radiation damage. The projection electron density maps at 77 K after the irradiation of 5.5 × 10^7^ Gy was consistent with that at ambient temperature after 4.3 × 10^5^ Gy [Figs. 5 ▸(*a*) and 5(*b*)]. In addition, the total irradiation dose of 1.1 × 10^8^ Gy was much smaller than the theoretically predicted upper limit to determine the 3D structure at a resolution of 141 nm at cryogenic temperature (Howells *et al.*, 2009 ▸).

After the tomography experiment, the toner particle was returned to the load-lock chamber and was kept at 298 K. Then, the particle was again transferred to the cryogenic pot and cooled to 77 K. Figs. 5 ▸(*c*) and 5(*d*) show the surface and internal structures of a warmed-and-cooled toner particle, respectively. The surface structures were similar to that obtained in the first tomography experiment (Fig. 3 ▸). However, in the internal structure, a void (or extremely low density) region appeared under the F2 surface [sections 3–4 in Fig. 5 ▸(*d*)]. As the 3D map of the first cryogenic experiment had no void regions (Fig. 4 ▸), the structural change probably appeared in warming the particle after the first data collection. Although the mechanism to produce the void structure is unknown, diffraction data collection at cryogenic temperatures may be essential to suppress the thermal diffusion of any quantum chemical changes of polymers caused by X-ray irradiation (Miura, 2000 ▸).

### 4.3. Three-dimensional reconstruction

In this study, we used the SIRT algorithm based on the back-projection (BP) method and not the three-dimensional phase retrieval (3D-PR) calculation, for the following reasons.

In our previous cXDI-TM studies of biological cells (Kobayashi *et al.*, 2018 ▸; Nakasako *et al.*, 2020 ▸), the BP-based reconstruction algorithm yielded 3D maps displaying smaller crystallographic *R*-factors than those of the 3D-PR calculations applied to the 3D reconstructed distribution of diffraction intensity. Furthermore, the 3D maps reconstructed by using the BP-based method are easier to interpret the cellular internal structures than those given by the 3D-PR calculation.

From a technical point of view, the 3D-PR calculation requires large computational costs both in memory and time. Generally, as the 3D-PR calculation has no experimental information on phase-angle of diffracted waves, more than 100 trial calculations are required to examine the correctness and the convergence of the calculation. For instance, when using the HPC supercomputer system at SPring-8, a single 3D-PR calculation takes more than two days for the 3D reconstructed structure amplitudes. In cXDI-TM experiments, as we intend to complete the 3D reconstruction within a few hours after the data collection, the use of 3D-PR calculation is non-realistic. In addition, as the 3D distribution lacked the diffraction amplitudes in the angular ranges from −78° and −90° and from +78° and +90°, these missed volume may cause artifacts in the 3D-PR calculation. Although the BP-based algorithm may also cause artifacts in the 3D reconstructed map, we will overcome the problem using the structure refinement as described in the next section.

### 4.4. Structure refinement

In this study, the structural analyses were performed temporarily at a resolution of 7.1 µm^−1^ (141 nm in real space). The distributions of three electron-density classes were visualized (Fig. 4 ▸). The constituent of class I was speculated with the help of powder diffraction. The constituents of classes II and III that still remain unknown may be estimated by extending the resolution beyond 25 µm^−1^.

The achievable resolution in the 3D reconstruction of a 4.8 µm-sized particle using the 313 diffraction pattern is estimated to be 48 nm by the following expression (Frank, 2006 ▸),
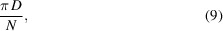
where *N* is the number of projection images of the particles in different orientations against the direction of the incident beam, and *D* is the size of the particle. However, in addition to the size of the computational resources, the following problems caused by SIRT reconstruction should be resolved.

**Figure 6:**
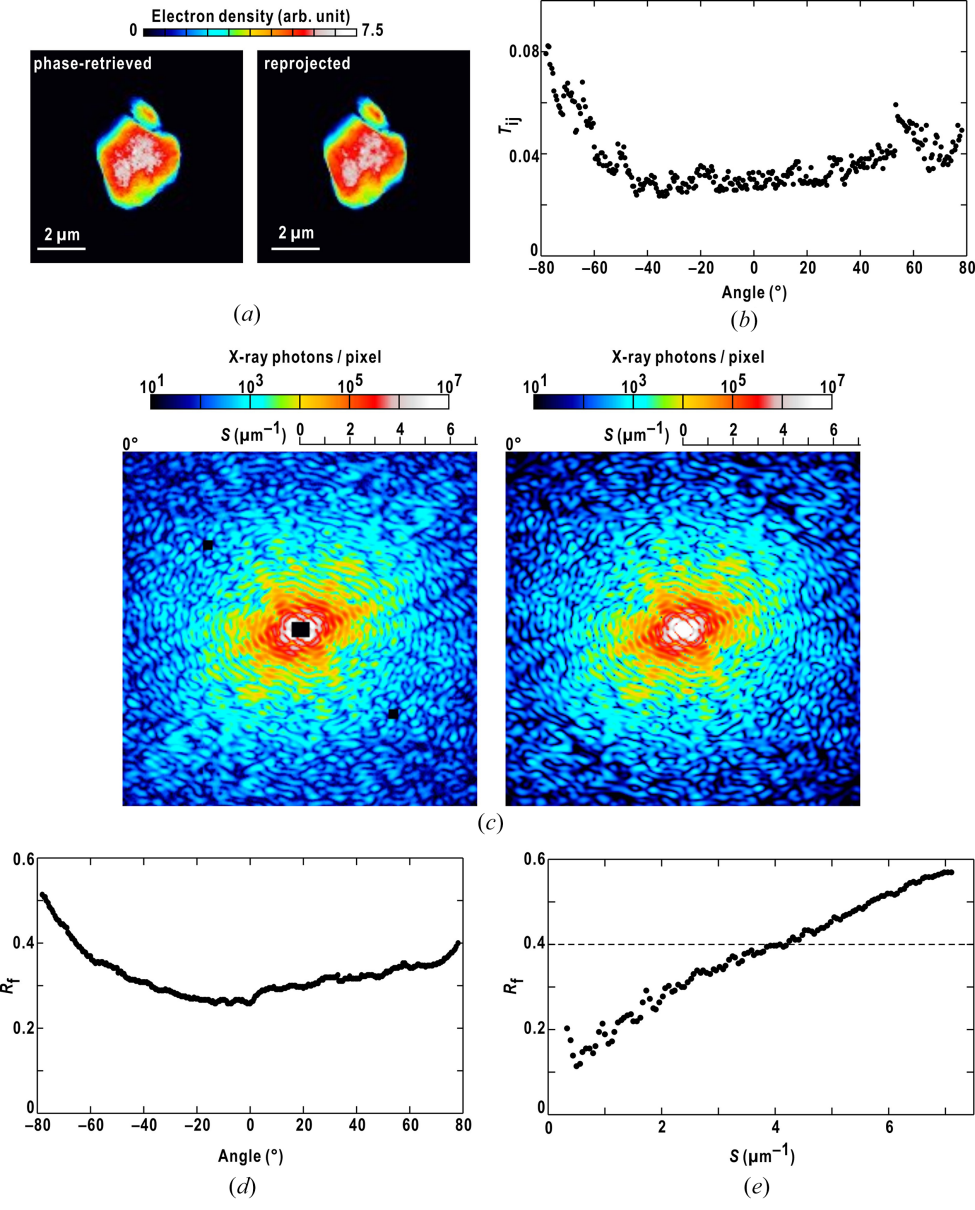
Comparison of the reconstructed 3D map with experimental data. (*a*) The PR map at 0° (left panel) and the map reprojected from the 3D map (right panel). (*b*) Similarity scores between the PR map and the reprojected map at each angle. (*c*) The observed pattern (left panel) and the pattern calculated from the reprojected map in panel (*a*) (right) at 0°. (*d*) Crystallographic *R*-factor at each angle. (*e*) Resolution-dependent variation of the crystallographic *R*-factor of the diffraction amplitudes from the 3D map against the observed ones.

In Fig. 6 ▸(*a*), fine structures that were absent from the PR map appeared in the map re-projected from the reconstructed 3D map. Such differences between the PR and re-projected maps are reflected quantitatively in the similarity scores [Fig. 6 ▸(*b*)]. In reciprocal space, the diffraction patterns calculated from the 3D map were similar to the observation up to 4.3 µm^−1^ [Fig. 6 ▸(*c*)]. This indicates that the 3D map approximated the shape of the toner particle and the long-range distance correlation. However, patterns beyond *S* > 4.3 µm^−1^ differed from each other. These differences resulted in inconsistencies in the diffraction amplitudes reflected in the *R*-factors [Figs. 6 ▸(*d*) and 6(*e*)].

Based on our experiences with protein crystallography, as preliminary phase-estimated maps display *R*-factors of 0.4–0.5, the 3D map obtained in this study may be at the level of the preliminary maps. It should be refined to explain the observed diffraction amplitudes. In protein crystallography, structure refinement simultaneously minimizes the *R*-factor in reciprocal space and the internal energy and/or deviation from the ideal stereochemistry of a protein model in real space (Murshudov *et al.*, 1997 ▸; Brünger *et al.*, 1998 ▸). However, to structurally refine the electron density distributions inside noncrystalline particles, target functions in real space should be developed. In addition, the 3D distribution of the diffraction amplitudes may be influenced by the center of confusion in the rotation stage, which is intrinsically present even after a highly meticulous manufacturing process. In protein crystallography, the center of confusion is determined through a post-refinement procedure (Rossmann *et al.*, 1979 ▸). Similar data processing may be necessary to provide diffraction amplitudes corrected with respect to the center of confusion.

## Supplementary Material

Gif movie of diffraction patterns in tomography experiment. DOI: 10.1107/S1600577525008008/yn5127sup1.gif
