# Supplementary figures and images for: Three-dimensional structure of printer toner visualized using cryogenic X-ray diffraction imaging tomography
Source: J Synchrotron Radiat. 2025 Oct 16;32(Pt 6):1491–502. doi: 10.1107/S1600577525008008 (PMC12591063; doi:10.1107/S1600577525008008)

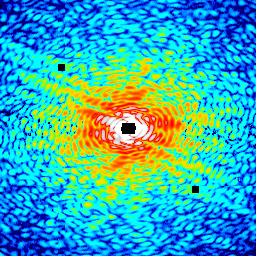

Supplement: Supplementary file 1 [file s-32-01491-sup1.gif]
